# Finsen Light

**Published:** 1902-09-27

**Authors:** 


Hibernicus writes:—"Among the 'Notes and News paragraphs there is a reference to this light being in use at the Royal Infirmary, Dublin; but this is inaccurate, as there is no public hospital or institution of that name in Dublin.

"The Finsen apparatus is, however, installed at the Royal City of Dublin Hospital, Upper Baggot Street, Dublin, having been provided there through the private generosity of one of the directors of that well-known Institution"

